# Control of monomeric Vo’s versus Vo clusters in ZrO_2−x_ for solar-light H_2_ production from H_2_O at high-yield (millimoles gr^−1^ h^−1^)

**DOI:** 10.1038/s41598-022-19382-3

**Published:** 2022-09-07

**Authors:** Yiannis Deligiannakis, Asterios Mantzanis, Areti Zindrou, Szymon Smykala, Maria Solakidou

**Affiliations:** 1 Laboratory of Physical Chemistry of Materials and Environment, Department of Physics, University of Ioannina, Ioannina, Greece; 2 Laboratory of Biomimetic Catalysis and Hybrid Materials, Department of Chemistry, University of Ioannina, 45110 Ioannina, Greece; 3 Institute of Engineering Materials and Biomaterials, Silesian University of Technology, 18a Konarskiego St, 44-100, Gliwice, Poland; 4 Institute of Environment and Sustainable Development, University Research Center of Ioannina, 45110 Ioannina, Greece

**Keywords:** Nanoparticles, Synthesis and processing, Photocatalysis, Characterization and analytical techniques

## Abstract

Pristine zirconia, ZrO_2_, possesses high premise as photocatalyst due to its conduction band energy edge. However, its high energy-gap is prohibitive for photoactivation by solar-light. Currently, it is unclear how solar-active zirconia can be designed to meet the requirements for high photocatalytic performance. Moreover, transferring this design to an industrial-scale process is a forward-looking route. Herein, we have developed a novel Flame Spray Pyrolysis process for generating solar-light active nano-ZrO_2−x_ via engineering of lattice vacancies, Vo. Using solar photons, our optimal nano-ZrO_2−x_ can achieve milestone H_2_-production yield, > 2400 μmolg^−1^ h^−1^ (closest thus, so far, to high photocatalytic water splitting performance benchmarks). Visible light can be also exploited by nano-ZrO_2−x_ at a high yield via a two-photon process. Control of monomeric Vo versus clusters of Vo’s is the key parameter toward Highly-Performing-Photocatalytic ZrO_2−x_. Thus, the reusable and sustainable ZrO_2−x_ catalyst achieves so far unattainable solar activated photocatalysis, under large scale production.

## Introduction

Sunlight energy storage to H_2_ via photocatalytic water splitting^1,2^ bears key advantages, such as high energy-storage capacity of H_2_ 141.6 kJ g^−1^ versus 0.46–0.72 kJ g^−1^ on Li-batteries^3^. Water photocatalysis to produce H_2_ is an inherently green-technology, fully compliant with circular economy^4^. Moreover, maximization of photocatalytic efficiency *in tandem* with minimized costs for industrial-scale production is mandatory. So far, most literature reports on high-performance photocatalysts (HPP) refer to nanomaterials with focus on performance optimization^5^. Except TiO_2_, industrial-scale and cost-effective production of HPP’s remains at its infancy.

Metal oxides can be HPP’s as long as they fulfill certain key criteria: H_2_ production and CO_2_ reduction, are favored by high conduction band (CB) semiconductors. Pristine zirconia (ZrO_2_) has one of the highest CB-edge energies among metal oxides E_CB_ =  − 1100 mV versus NHE. However, its band-gap energy E_g_ > 5 eV requires highly energetic photons, i.e., λ < 225 nm, which is prohibitive for solar-light harvesting. In this context, pristine ZrO_2_ despite its established uses in technology, e.g. as refracting coating^6^, thermal coating^7^, gas-sensing^8^, fuel cells^9^, Water–Gas-Shift reaction^10^, so far, has not been established as a high-performance photocatalyst. Historically, Sayama and Arakawa^11^ were the first to observe photocatalytic performance of pristine ZrO_2_ reporting a rather symbolic H_2_-production yield 72 μmol g^−1^ h^−1^ under X-ray irradiation.

Strategies to ameliorate photocatalytic ZrO_2_ activity, can include: (i) heteroatom insertion^12–17^ into ZrO_2_ crystal, or (ii) Oxygen-defects’ creation^18–23^. The influence of heteroatoms in ZrO_2_ has been investigated^12–17^, in photocatalytic dye degradation or O_2_ evolution. Cerium-doped ZrO_2_ (Ce-ZrO_2_) can be photoactive in visible λ > 420 nm light^12^. Erbium-doped ZrO_2_^14^ allowed band-gap tuning towards solar photons, which contributed to improved Methylene-Blue degradation^13^. Nitrogen^15^ and carbon^17^ 2p-orbitals can enhance photocatalytic dye degradation via generation of intra-bandgap states. However, in all aforementioned cases, the reported photocatalytic performances, despite improvement, remain by far inferior versus benchmark photocatalysts, such as TiO_2_ which routinely achieves H_2_ photoproduction of the order of several (millimoles H_2_ g^−1^ h^−1^) in typical lab set ups^24^. So far, the only ZrO_2_-based photocatalyst passing the threshold of (millimoles g^−1^ h^−1^) is 2.12 mmol H_2_ g^−1^ h^−1^ by a N-doped ZrO_2_^25^.

Without heteroatom doping, generation of reduced states in reducible metal-oxides, such as TiO_2_, are decisively beneficial for H_2_ photo yield. Examples include the work of Mao et al.^23^, Naldoni et al.^26^ and our Flame Spray Pyrolysis (FSP)-made black TiO_2−x_^27^. ZrO_2_ is a notoriously non-reducible oxide^28,29^, since introduction of Vo’s into the ZrO_2_ lattice is not favored energetically^8,15–20^, thus specific synthesis methods are needed to achieve reduction of the ZrO_2_ lattice. To this direction, the most significant performance has been reported by Sinhamahapatra et al.^22^ and Zu et al.^30^, where defect-rich ZrO_2−x_ has achieved production of ~ 0.5 mmolg^−1^ h^−1^ H_2_ in both cases, however, this is still significantly below that of TiO_2_^26,31^.

Regarding material synthesis, none of the so far reported ZrO_2−x_ synthesis methods were designed to be scalable at industrial level. Specifically, previous ZrO_2−x_ synthesis methods include sol-gel^19^, hydrothermal^20^, high pressure/temperature processing^18,21^. More efficient methods able to overcome the non-reducibility of ZrO_2_ are magnesiothermic^22^, titaniothermic^23^ and lithiothermic reduction^30^ where an elementary heterometal M^0^ atom, i.e. Li^0^, Ti^0^, Mg^0^, is used to reduce ZrO_2_ and create the desired Vo at high yield. However, all aforementioned synthesis routes include multiple steps and do not always allow facile/reproducible control or tailoring of Vo placement and populations. Particularly, the methods which require heterometal contact on ZrO_2_ surface e.g. magnesiothermic, rely on harsh acid washes for removing the leftover hetero-metal oxide, something that mounts questions on how this may impact the state of the catalyst itself^22^.

Herein we have developed a one-step Flame Spray Pyrolysis (FSP) process for synthesis of solar-light active nano-ZrO_2−x_ via engineering of lattice-vacancies, Vo. FSP is eminently suited for synthesis of high crystallinity nano-ZrO_2_^32,33^, however, the synthesis of ZrO_2−x_ has not been reported by FSP. In principle, ZrO_2_ can possess two families of reduced states: (i) reduced Zr^3+^ centers, and (ii) oxygen vacancies not-located on Zr atoms (Vo’s). Over last decades, Giamello’s group^34^ has provided valuable insights into the complexity of these reduced states. Using Electron Paramagnetic Resonance (EPR) spectroscopy, in combination with quantum chemical calculations^34^, they prove that Zr^3+^ centers can create extra energy states right below the E_CB_ of pristine ZrO_2_, (~ 4.5–5.0 eV). Based on all existing evidence, these Zr^3+^ centers are expected to have little effect on the photocatalytic activity of zirconia^35^. On the other hand, Vo’s can create mid-gap states^34^ but their role in photocatalytic H_2_ evolution has not been explored systematically. Herein, using FSP we have produced libraries of ZrO_2−x_ nano catalysts with varying concentrations of Vo’s and identified the optimal configuration, towards high photocatalytic H_2_-production efficiency from H_2_O. Specific aims of the present work were: (i) to develop a novel industrial-scale FSP method for one-step synthesis of nanosized ZrO_2−x_ with controllable population and placement of the O-vacancies (Vo’s). No heteroatoms were used. (ii) to optimize the ZrO_2−x_ for highly efficient solar light H_2_ production, well beyond the current state of the art, i.e., well-above the threshold mmol g^−1^ h^−1^, (iii) to provide a comprehensive understanding of the physicochemical role of Vo, with emphasis on monomeric versus clusters of Vo’s related to the photocatalytic properties. We present a novel anoxic-FSP process that allows *in-situ* formation of Vo’s during the primary particle formation step. Using solar photons, the optimal nano-ZrO_2−x_ can achieve milestone H_2_-production yield, > 2400 μmolg^−1^ h^−1^ which is the closest so far to high photocatalytic performance benchmarks. We demonstrate that optimal nano-ZrO_2−x_ can be achieved by controlling the monomeric Vo versus clusters of Vo’s by two routes either during the FSP synthesis or via a short post-FSP oxidation process.

## Results

### Synthesis of nano ZrO_2−x_ by anoxic-flame spray pyrolysis

**Figure 1:**
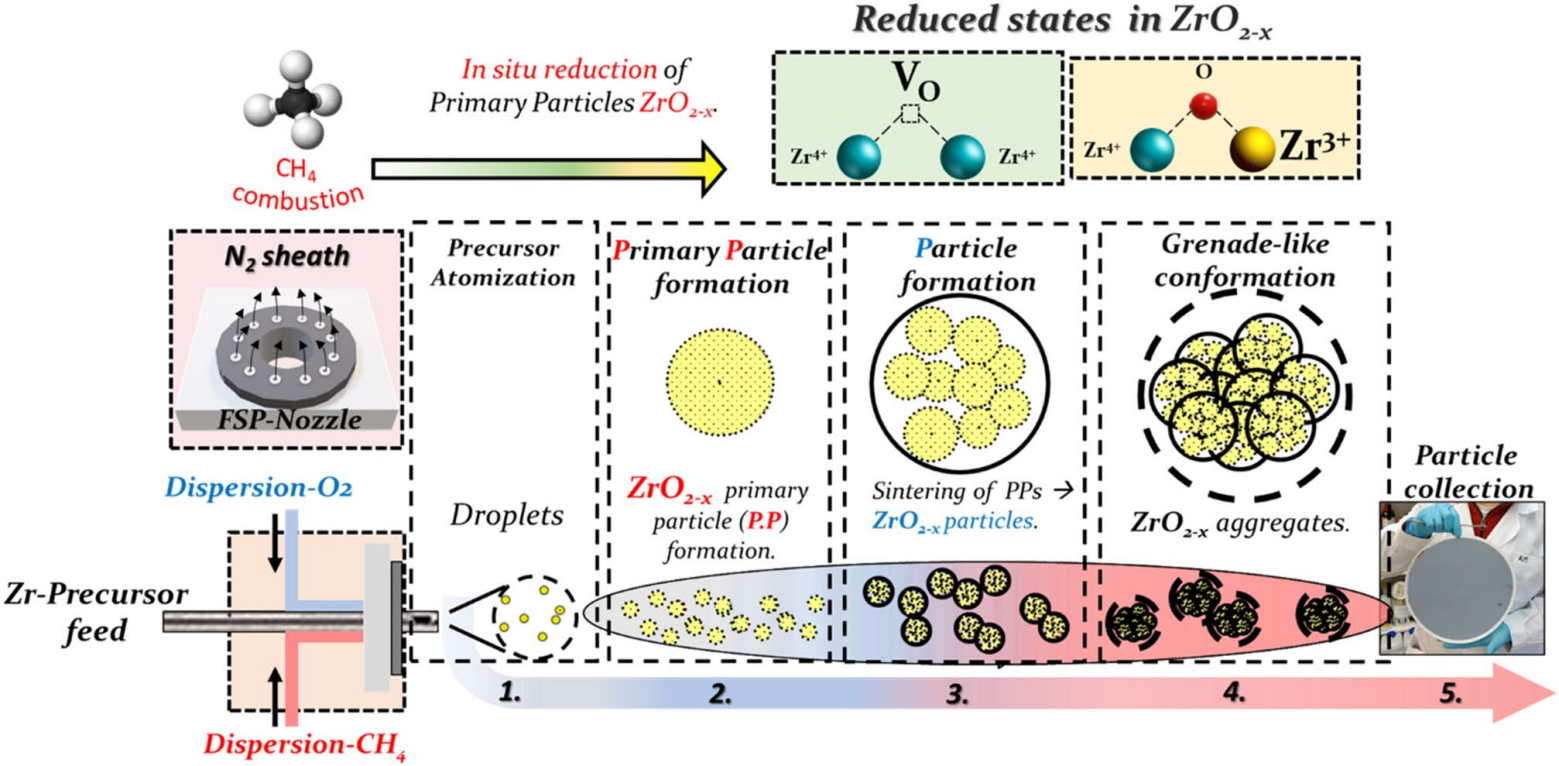
One-step FSP process for ZrO_2−x_ production. The photo at right shows as-produced 500 mg of [1.3/1.7] ZrO_2−x_ particles.

The concept of the novel anoxic-FSP process is outlined in Fig. 1. It consists of a single-nozzle FSP reactor with enclosed flame, where a mixture of Dispersion-gas [O_2_ and CH_4_] is used to create a reducing reaction atmosphere. In FSP-process^35^, ZrO_2_ particles are formed in three stages (Fig. 1). First, Zr-precursor droplets are sprayed by the FSP nozzle and combusted to generate the primary particles (PP)^35^. Then, primary particles evolve in the high-temperature area of the flame, i.e. up to 2800 K and form nanometric ZrO_2_ particles via sintering of PP’s^35^. In classical-FSP, used in the majority of lab studies and industry, pure O_2_ is used as dispersion gas through the spray nozzle, to form the droplets and primary particles. For example, by adjusting the combustion stoichiometry^35^ ratio P/D = [fuel/dispersion O_2_] = 3/3, we obtain fully oxidized, pristine ZrO_2_. In our anoxic-FSP, combustion of CH_4_ in the dispersion gas creates reducing agents which, as we show herein, can reduce the primary Zr-particle via formation of Oxygen vacancies (Vo). We also have considered the possibility of the formation of Zr-Hydride states^36^, however, none of our data support this, thus we do not discuss it further.

**Figure 2:**
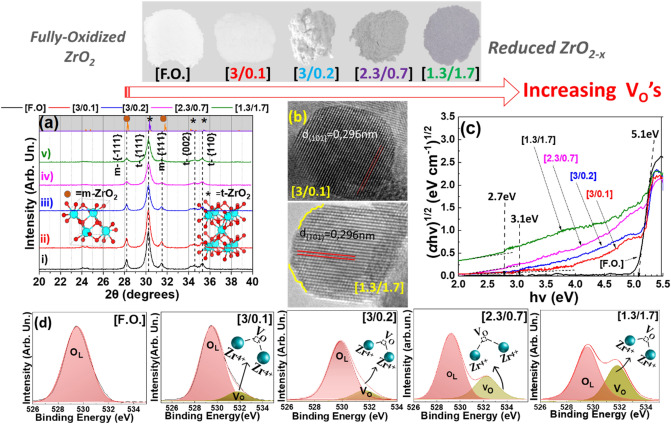
(**a**) XRD of FSP-made ZrO_2*−*x_ materials, (**b**) TEM images for [3/0.1] and [1.3/1.7] materials, (**c**) Kubelka–Munk plots DRS UV–Vis spectra. (**d**) XPS spectra of F.O., [3/0.1], [3/0.2], [2.3/0.7] and [1.3/1.7]. *Top-Inset*: Photos of the nanopowders.

In this way, we have produced a library of ZrO_2−x_ nanoparticles of varying Vo concentrations, see photos in Fig. 2. Herein we have codenamed the particles according to the dispersion [O_2_/CH_4_] ratio used, (see full details in Table S1 in SI). For example, the material codenamed [3/0.1] has been synthesized using a dispersion gas-mixture of [O_2_ flow = 3.0 lt min^−1^ and CH_4_ flow = 0.1 lt min^−1^]. Pristine ZrO_2_ is codenamed F.O. for Fully Oxidized, while material [1.3/1.7] was the most reduced. As shown in Fig. 2, going from pristine ZrO_2_ (F.O.) towards more reduced particles, i.e. [3/0.1] to [1.3/1.7], induced a change of particle color from crispy-white to pale grey/yellowish [3/0.1] and dark-gray for [1.3/1.7] material. XPS data (Fig. 2d) shows a progressive increase of Vo’s, detected by their characteristic signal at 532 eV^37,38^. We have verified that no-carbon deposition is evidenced by Raman data, (Supplementary Fig. S1), thus the observed color changes in the ZrO_2−x_ materials (Fig. 2), are assigned exclusively to the formation of Vo’s via the anoxic-FSP process. According to XPS, Fig. 2d increased dispersion of the CH_4_ in the FSP process, promotes the formation of Vo’s. No Zr^3+^ states are resolved in Zr-XPS data, i.e., only the Zr^4+^ doublet was detected (181.9 eV, 184.2 eV) (Supplementary Fig. S2a–d).

The DRS-UV/Vis data, (Fig. 2c) show a progressive alteration of the band gaps as evidenced by the absorbance at intra-gap energies of 2.5 to 4.5 eV. All materials exhibit the primary bandgap 5.1 eV (243 nm) typical for monoclinic-ZrO_2_^18,20^. Fully oxidized ZrO_2_ exhibited only the primary bandgap at 5.1 eV with no mid-gap states. Slightly-reduced [3/0.1] exhibited an additional absorbance tail band extending to ~ 3.1 eV. Low concentration of Vo’s, created by 0.1 Lmin^−1^ CH_4_, (see XPS data Fig. 2d) [3/0.1], allows absorbance of photons near the middle of the primary bandgap, and creates a pale-yellow color, see photo Fig. 2. Increasing dispersion CH_4_ (materials [3/0.2], [2.3/0.7], [1.3/1.7]) causes a monotonous enhancement of the intra-band absorbances and grey-color intensification (Fig. 2c). Experimentally^18,20,22,23,30^ and theoretically^29,34^ the intra-band absorbances in the range of 2.8–3.5 eV, can be assigned to electrons being injected from the VB_maximum_ to approximately the middle of the gap, where extra DOS are made available through the introduction of Vo’s. For example, by removing one oxygen atom, a doubly occupied (diamagnetic) F-center^39^ can be created inside the bandgap ZrO_2−x_ at 3.3 eV^39^.

### Structural characterization

**Figure 3:**
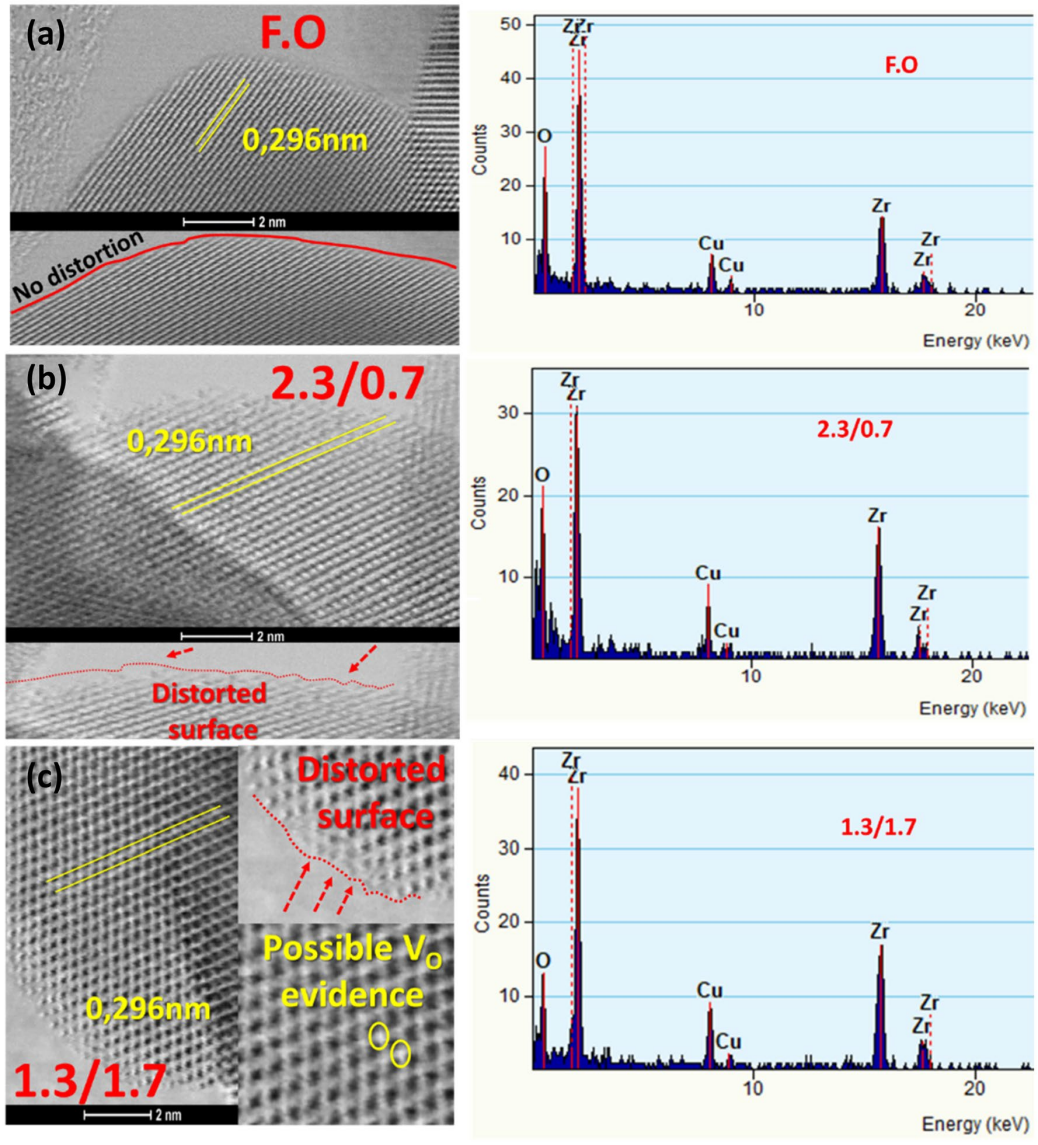
STEM images and EDX spectra of (**a**) F.O. (**b**) 2.3/0.7 and (**c**) 1.3/1.7 materials.

XRD (Fig. 2a) shows that our FSP-made Zr-particles consisted of monoclinic (*m*-ZrO_2_ in Fig. 2a) and tetragonal (*t*-ZrO_2_ in Fig. 2a) phases, at a ratio [t-ZrO_2_]: [m-ZrO_2_] = 4:1 and particle sizes 22–29 nm (monoclinic) to 16–20 nm (tetragonal phase) (Table S2). At ambient P, T, ZrO_2_ has monoclinic (m) structure with Zr^4+^ atoms sevenfold coordinated by O-anions (space group P2_1_/c). TEM images, (Figs. 2b, 3), show that all ZrO_2−X_ nanomaterials have quasi-spherical shapes, with highly crystalline Miller planes *t*-{101}. The more reduced ZrO_2−X_ nanomaterials show some degree of surface distortion, see Fig. 2b. STEM images (Fig. 3a–c) indicate that the particles retain a high degree of crystal quality even at the more reduced ones. In some cases, formation of vacancies could be evidenced in the STEM images, (Fig. 3c). EDX data (Fig. 3) confirm a strong decrease of the Oxygen/Zr ratio in the more reduced [2.3/0.7] and [1.3./1.7] materials. BET analysis (Table S2) shows progressive SSA-decrease upon increase dispersion-CH_4_, attributed to increased aggregation of the particles at increased CH_4_, i.e. methane creates hotter flames i.e. methane heat of combustion = 50–55 MJ/kg (https://webbook.nist.gov/chemistry/).

**Figure 4:**
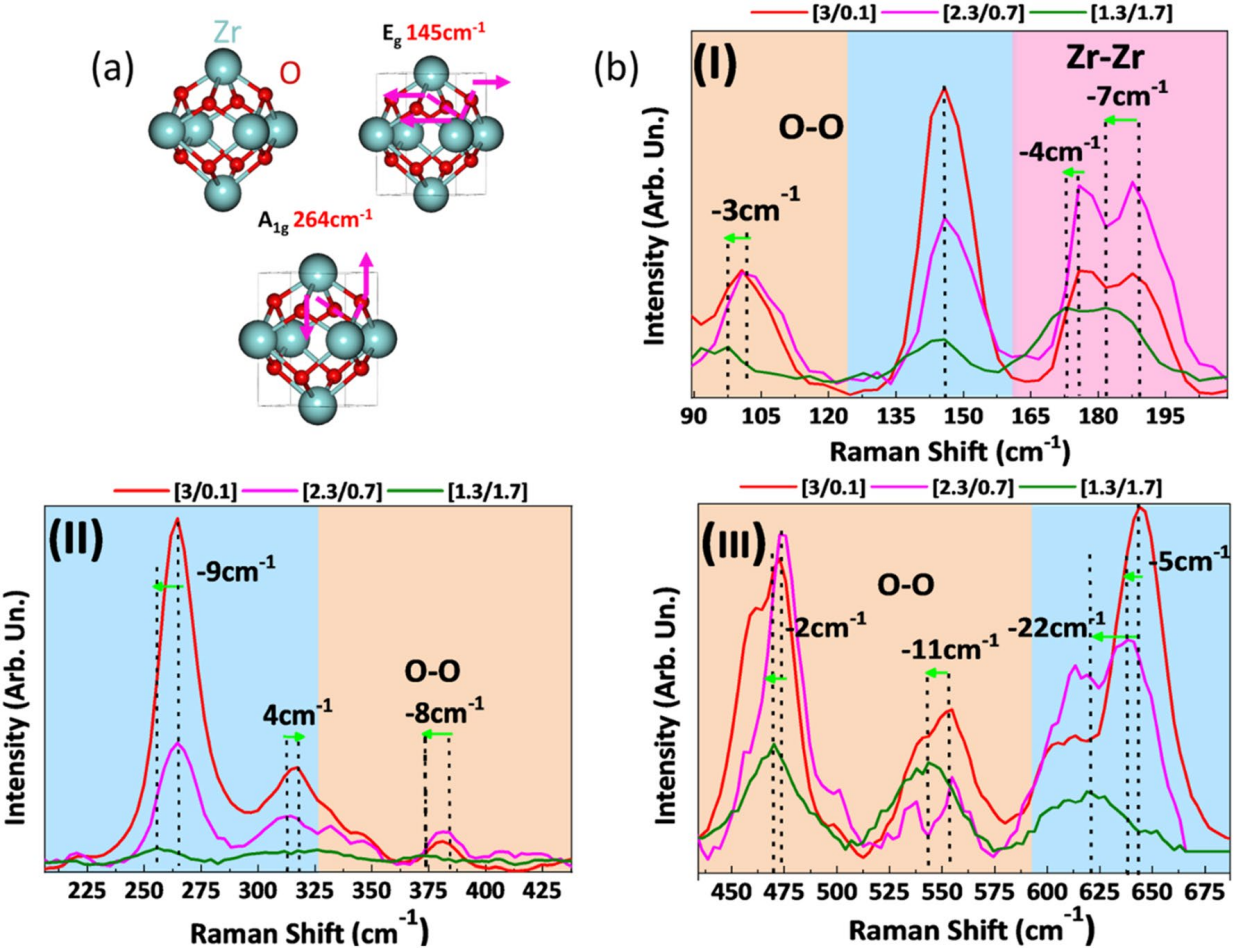
(**a**) Unit cell of ZrO_2_with two indicative t-ZrO_2_ lattice vibrations and (**b**) Raman downshifts in reduced ZrO_2−x_.

Raman spectra (Fig. 4b) exhibit the vibrational modes from both monoclinic and tetragonal crystal phases^40–43^ (Fig. S4 and Table S3) and absence of carbon peaks (Fig. S1). In ZrO_2−x_ materials, certain Raman modes are shifted (see Fig. 4b(I–III) and Table S4). More particularly, material [3/0.1] exhibits upshift of + 4 cm^−1^ at 313 cm^−1^ mode (Table S4). Material [2.3/0.7] exhibits downshifts of − 5 cm^−1^, − 2 cm^−1^, − 4 cm^−1^ at 313 472 cm^−1^, and 643 cm^−1^ respectively (Table S4). Material [1.3/1.7] exhibits downshift of up to 22 cm^−1^. Such downshifts can be attributed to tensile stress^44^ effectively lengthening the bonds, see Fig. 4a, i.e. due to loss of oxygen atoms from the lattice. Raman downshifts prevail in the *t*-ZrO_2_ phase; thus, the tetragonal phase is more responsive in the reductive atmosphere in anoxic-FSP. This can be explained by the existence of two different/non-equivalent Zr-O bond conformations in *t*-ZrO_2_^44^ corresponding to 4-coordinated Zr(O_4f_), and 3-coordinated Zr atoms (O_3f_) respectively^44^. For comparison, the *t*-ZrO_2_ matrix consists solely of O_4f_ Zr atoms while the m-ZrO_2_ matrix consists of both O_4f_ and O_3f_^44^. Accordingly, the present Raman data indicate that removal of oxygen from the ZrO_2_ matrices is also non-equivalent, thus is easier to extract oxygen from an O_4f_ site rather than an O_3f_ site by 0.1 eV^22^, therefore it is easier to reduce *t*-ZrO_2_.

### Photocatalytic H_2_ production at (millimole gr^−1^ h^−1^)

**Figure 5:**
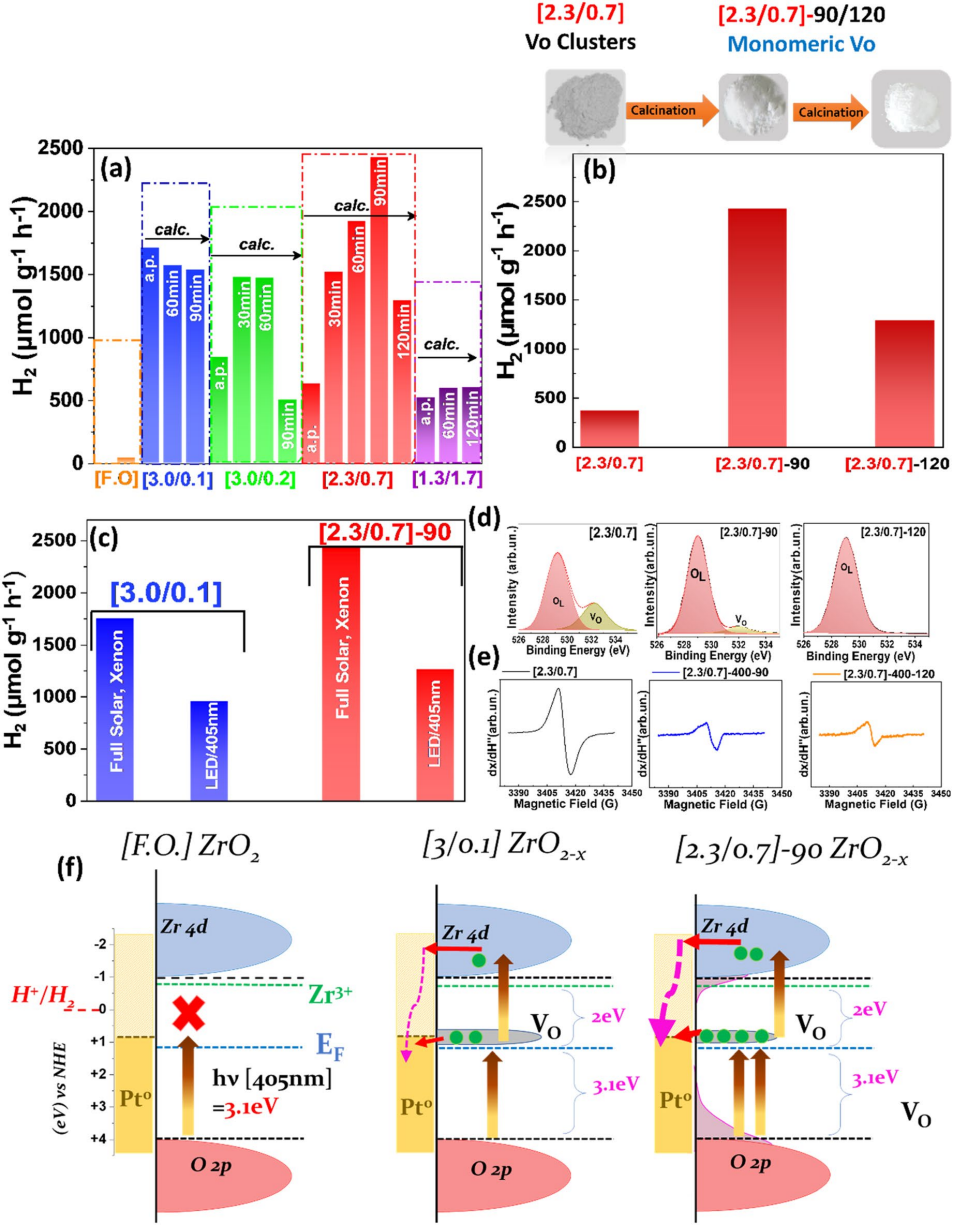
(**a**) Photocatalytic H_2_ production from H_2_O, by as-prepared and oxidized ZrO_2−x_ catalysts under full-solar Xenon-light irradiation. (**b**) H_2_ production by [2.3/0.7] ZrO_2−x_ catalyst under various oxidation times (400 °C). (**c**) Comparison of photocatalytic H_2_ production by the best performing ZrO_2−x_ catalysts under full-solar Xenon-light versus visible 405 nm LED irradiation. (**d**) XPS spectra, (**e**) EPR spectra, indicating the evolution of the Vo’s under oxidation of [2.3./0.7] ZrO_2−x_. (**f**) Schematic DOS configuration and excitation under visible 405 nm, see full DFT data in S.I. Fig. S10.

Figure 5a presents the photocatalytic H_2_ production from H_2_O, for all our ZrO_2−x_ materials, under Xenon-illumination. In each panel in Fig. 5a the as-prepared photocatalysts are marked as “a.p”. The time indication in each bar, refers to the post-FSP oxidation-time at 400 °C (see also XRD data in Fig. S3 in S.I.). First, we discuss the as-prepared ZrO_2−x_ materials i.e., see the first bar in each column group in Fig. 5a. Pristine, (F.O.) ZrO_2_ was practically non-photoactive in H_2_ production, with a yield of 20 μmol g^−1^ h^−1^.

In contrast, a slightly reducing FSP atmosphere, i.e., as-prepared [a.p. 3/0.1], enables an impressive amelioration of H_2_ evolution of 1700 μmol g^−1^ h^−1^. This demonstrates that anoxic-FSP can provide as-prepared ZrO_2−x_ material exhibiting millimoles per gram per hour H_2_ production. Further increase of O_2_/CH_4_ ratio impacted negatively the H_2_ photogeneration with a tendency towards a steady production near 500 μmol g^−1^ h^−1^ of H_2_ for the highly reduced as-prepared materials (see the first bar in each group in Fig. 5a). Soft oxidation by calcination at 400 °C under ambient O_2_, exerted a dramatic influence on the H_2_-photoproduction: a characteristic bell-shaped dependence was observed for the [3.0/0.2] and [2.3/0.7] materials. The optimum oxidation time was 90 min for [2.3/0.7] ZrO_2−x_ which, achieved a remarkable yield of 2428 μmol H_2_ g^−1^ h^−1^. The best performance of the [3.0/0.2] ZrO_2−x_ material was 1500 μmol H_2_ g^−1^ h^−1^ (Fig. 5a). Table S5 summarizes a comparison of FSP-ZrO_2−x_ versus other pertinent ZrO_2−x_ materials reported in the literature (see also Fig. S7).

The catalyst with the higher H_2_ yield, [2.3/0.7]-90, is highly recyclable, (Fig. S5a), retaining 100% of its activity after two reuses and > 96% after four reuses. XRD (Fig. S5b), shows that the [2.3/0.7]-90 crystal remains intact after 4-uses. Concurrently, DRS-UV/Vis (Fig. S5c), demonstrates that its light-absorbance profile remains also intact. As we discuss hereafter, optimization of monomeric Vo-concentration is determinant for photocatalytic activity. In [2.3/0.7]-90, the monomeric Vo’s is optimized, see EPR and XPS data in Figs. 5d,e, and S6a,b see also the trends in XPS, EPR for [2.3/0.7] in Fig. 5d,e. After photocatalytic use of [2.3/0.7]-90 material, the monomeric vacancies are not altered, neither the ZrO_2−x_ crystal. Thus, the ZrO_2−x_ provide a robust reusable photocatalyst.

## Discussion

The data in Fig. 5a demonstrate that there are two options to achieve high-photocatalytic performance ZrO_2−x_ materials: either (i) to be prepared at low O_2_/CH_4_ ratio e.g. [3.0/0.1], or (ii) after a soft post-FSP oxidation of more reduced materials e.g. [2.3/0.7]. Very-high O_2_/CH_4_ ratio [1.3/1.7] results in highly-reduced ZrO_2−x_ which cannot be improved by post-FSP oxidation.

**Figure 6:**
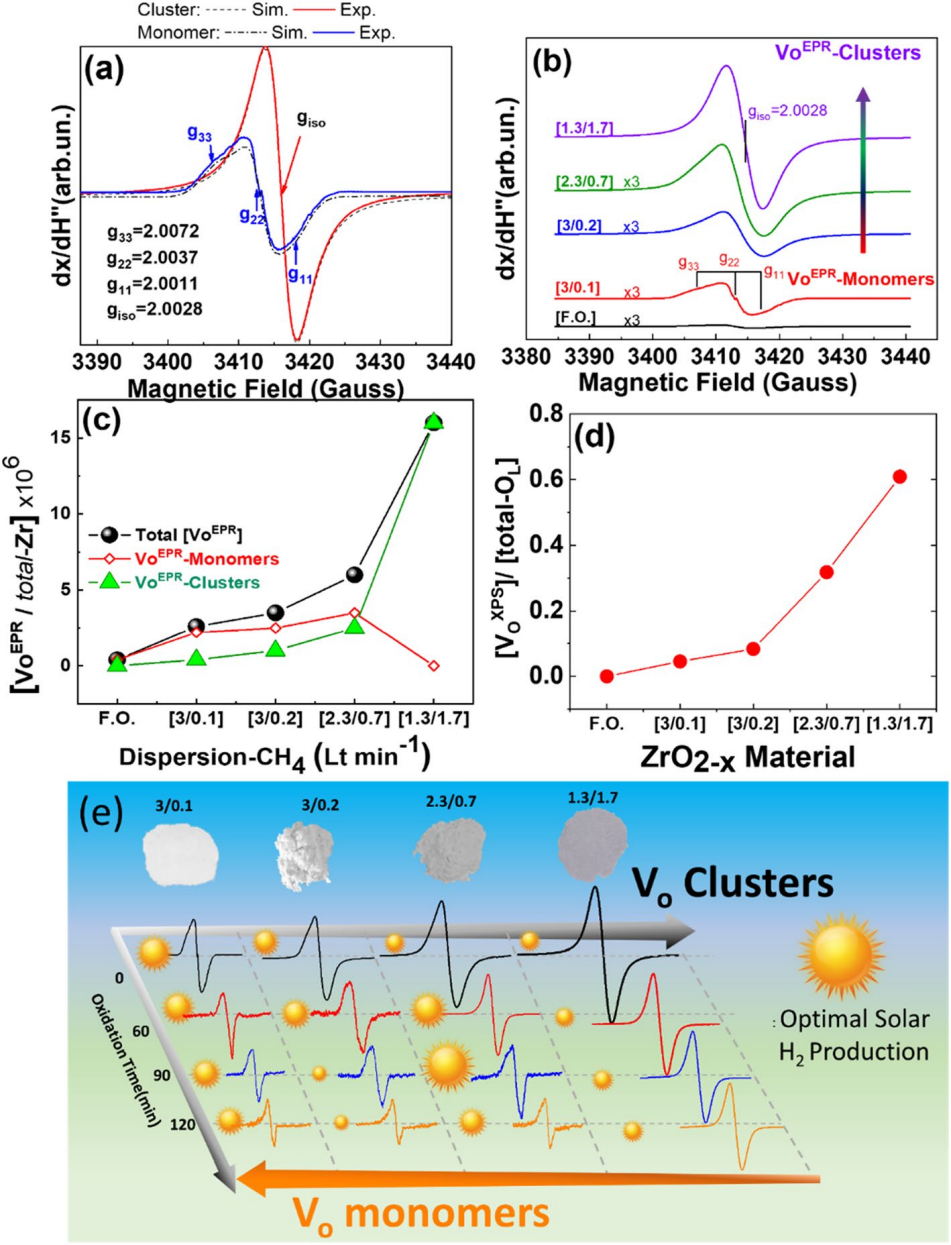
(**a**) Experimental (solid-lines) and theoretical EPR (dashed-lines) spectra of Vo’s formed in as-prepared [3/0.1] ZrO_2−x_ (blue, monomeric Vo’s) or [1.3/1.7] ZrO_2−x_ (red, Vo clusters) (**b**) Experimental EPR spectra, showing the progressive transition from pure-monomeric Vo’s in [3/0.1] ZrO_2−x_ towards Vo-clusters in [1.3/1.7] ZrO_2−x_ (**c**) Ratio of EPR detectable oxygen vacancies V_O_^EPR^ over total Zr-atoms present in each ZrO_2−x_ material (**d**) Ratio of Vo^XPS^ determined by XPS over total oxygen atoms in the lattice. (**e**) Control of (Monomeric Vo/Vo Cluster) ratio in as-prepared ZrO_2−x_ or via post-FSP oxidation, allows optimization of the photocatalytic H_2_ production. The size of the sun-symbol exemplifies the H_2_ production efficiency.

The origins of these trends can be understood by considering the types and populations of the Vo’s in ZrO_2−x_. Figure 5b,d,e indicate a correlation of the H_2_ production rates for the [2.3/0.7] ZrO_2−x_ catalysts, with the Vo’s detected by XPS, Fig. 5d, and EPR Fig. 5e. The XPS and EPR data show a clear decrease in the population of Vo’s of the [2.3/0.7] material upon oxidation at 400 °C. Importantly, theoretical simulation of the EPR signals, Fig. 6, allows distinction between Vo-clusters versus monomeric Vo’s. For example, in [a.p. 2.3/0.7] Vo clusters prevail, while in [3.0/0.1], monomeric Vo’s prevail, as we can see from percentages in Fig. 6c. Oxidation progressively eliminates Vo clusters towards monomeric Vo’s. This is also evident from the progressive elimination of the deep-grey color, and the changes in the UV–Vis spectra (Fig. S8 in SI). Taking this information into account, the bell-shaped H_2_ production trend in Fig. 5b, indicates that a high concentration of O-vacancies, forming Vo clusters, is detrimental to the photocatalytic activity of ZrO_2−x_. Fewer Vo’s are better suited for optimal photocatalytic activity (see trend in Fig. 5a, for [2.3/0.7]). However, further oxidation of Vo’s tends to delimit the photoactivity. This teaches us that a quantitative control of the Vo’s clusters versus monomeric Vo is necessary, to achieve highly-performance photocatalytic ZrO_2−x_, see full trend in Fig. 6e. We consider that this factor was also of pertinence in the magnesiothermically reduced ZrO_2−x_ materials^22^. Although not noticed by these authors^22^, inspection of their EPR spectra, shows that these correspond to Vo clusters, which concurs with their limited H_2_ production of 506 μmol H_2_ g^−1^ h^−1^, resembling our as-prepared [2.3/0.7] material.

The data under visible 405 nm LED irradiation (Fig. 5c), indicate that significant part of photocatalytic H_2_ production, at least 70% versus the solar-light photons, can be excited by visible 405 nm photons (3.1 eV). Taking into account the DRS-UV/Vis data, (Fig. 2c) this can be attributed to the occurrence of the mid-gap states, (Fig. 5f). Theoretical DFT calculations (Fig. S10) show that in ZrO_2−x_, few oxygen vacancies can create mid-gap states, located at energy distances around 3.0 eV^12,45^ from both the VB-top and ~ 2.0 eV from the CB-bottom. Thus, in ZrO_2−x_ the 3.1 eV photons (405 nm LED) are able to excite two consecutive electron transitions, (Fig. 5f). Increased anoxicity, i.e., as in material [2.3/0.7], enhances the DOS band-tailing (see S.I. Figs. S10 and 5f). This would increase the probability of two-photon electron photoexcitation via VB → Vo, and Vo → CB. These electrons are favorably transferred to the Pt particles, which act as electron collectors i.e. work function of Pt, φ =  + 0.9 eV versus NHE^46^, is favorable for acceptance of electrons from the highly-excited electrons in the CB.

### Quantitate analysis of V_O_-clusters versus monomeric Vo’s, by EPR and XPS

Numerical EPR simulations, dashed lines Fig. 6a, show that monomeric Vo’s were characterized by an inhomogeneous line-shape with linewidth $\Delta H_{monomer} = \left( {9.4 \pm 0.1} \right){\text{Gauss}}$ and a rhombic g_monomer_-tensor, (Table S5). Vo clusters are characterized by a Lorenz line-shape and $\Delta H_{cluster} = \left( {4.6 \pm 0.1} \right){\text{ Gauss}}$ and isotropic g_cluster_-tensor, (Table S6).

The structural significance of this is: for isotropic EPR signals with low g-anisotropy, a Gaussian line-shape is the fingerprint of the so-called *in*homogeneously-broadened S = 1/2 states^47,48^ which is indicative of isolated Vo’s, with no-interactions^47,48^. Physically, this indicates that in the ZrO_2−x_ particles produced under low CH_4_-flow e.g. [3/0.1], monomeric isolated Vo^EPR^ centers. The Lorentz line-shape of the Vo clusters indicates that it originates from Vo centers with spin-exchange and/or fast dipolar interactions. In ZrO_2−x_, this Lorentz line-shape indicates formation of Vo-clusters upon increasing dispersion-CH_4_.

Comparing the XPS data for surface-Vo’s (Fig. 6d) versus the quantitative data for total-paramagnetic Vo’s, (Fig. 6c and Table S7 in SI), derived from the EPR spectra (Fig. 6b) and their deconvolution in monometer/clusters (see example for [1.3/1.7] in Fig. S9 in SI), we notice a correlation: highly reduced ZrO_2*−*x_, have higher surface-Vo’s, and total paramagnetic Vo’s. Figure 6e provides an overview plot, which shows that optimization of H_2_ photocatalysis can be achieved via optimization of V_O_-Clusters versus monomeric Vo’s by two routes: (i) control of FSP anoxicity, or (ii) by soft post-FSP oxidation. This demonstrates that our novel anoxic-FSP process allows facile synthesis of solar-light active nano-ZrO_2*−*x_ via engineering of lattice-vacancies, Vo. Control of monomeric Vo versus clusters of Vo’s is the key-parameter toward Highly-Performing-Photocatalytic ZrO_2*−*x_. The anoxic-FSP process presented here, should be easily adaptable to existing industrial-scale FSP reactors. This offers an efficient technology that can be adopted in the future and provide new tools for the design of other families of photoactive nanomaterials via control of oxygen vacancies.

## Methods

### Synthesis of nanomaterials by FSP

Precursor solution was prepared by dissolving Zirconium (IV) Propoxide (70 wt% in 1-propanol) in xylene and acetonitrile in a 2.2/1.0 ratio at a concentration of 0.25 M. Then, the solution was fed through a capillary at 3 ml min^−1^ and dispersed to a self-sustained oxygen/methane (4–2 L min^−1^) pilot flame to initiate combustion. An important distinction must be made which leads to the innovation of the presented work. ZrO_2−x_ materials were prepared by modifying the dispersion feed. While keeping the 3 ml min^−1^ dispersion constant, methane gas (CH_4_) was fed along with the traditional dispersion gas (O_2_). The resulting high temperatures and hydride formation through the decomposition of methane lead to the formation of ZrO_2−x_. Furthermore, the protocol of methane injection ensures the formation of bulk defects as the particle is influenced at its early stages of flight, at the primary particle stage. Finally, the pressure drop was fixed at 1.5–2.0 bar, and an additional 10 L min^−1^ O_2_ sheath was used to aid in particle collection which was made possible by a vacuum pump (Busch V40) and by a glass microfiber filter (GF 6 257, Hahnemühle, Dassel, Germany).

### Characterization techniques

Powder X-Ray Diffraction (XRD) data were collected at room temperature using a Bruker D8 Advance 2theta diffractometer with copper radiation (Cu Ka, λ = 1.5406 Å) and a secondary monochromator operating at 36 kV and 36 mA. Crystal size is calculated by the Scherrer formula. X-Ray photoelectron spectroscopy (XPS) data were collected by a surface analysis ultrahigh vacuum system (SPECS GmbH) equipped with a twin Al–Mg anode X-ray source and a multichannel hemispherical sector electron analyzer (HSA Phoibos 100). The base pressure was 2 − 5 × 10^−9^ mbar. A monochromatized Mg Kα line at 1253.6 eV and analyzer pass energy of 20 eV were used in all XPS measurements. The binding energies were calculated with reference to the energy of C_1s_ peak of contaminant carbon at 284.5 eV. The peak deconvolution was calculated using a Shirley background.

Raman HORIBA-Xplora Plus spectrometer, equipped with an Olympus BX41 microscope. A 785 nm diode laser was used as an excitation source, and the laser beam was focused on the sample with the aid of the microscope. Before measurement, each powder material was softly pressed between two glass plates to form a pellet-like structure. Brunauer–Emmett–Teller (BET) adsorption–desorption isotherms were recorded at 77 K using a Quantachrome NOVA touch LX^2^. Outgassing was performed at 80 °C for 5 h under vacuum, before the measurements. The absorption data points in the relative pressure P/P_o_ range of 0.1–0.3 was used to calculate the specific surface area (SSA).

### Electron paramagnetic resonance spectroscopy

The X-band electron paramagnetic resonance (EPR) spectra of ZrO_2_/ZrO_2−x_ materials were recorded with a Bruker ER200D spectrometer at 77 K, equipped with an Agilent 5310A frequency counter. The spectrometer was running under a custom-made software based on LabView. Adequate signal-to-noise ratio was obtained after 15–20 scans, with a microwave power fixed at 20 mW. The EPR instrumental conditions were as follows: microwave frequency = 9.53 GHz and modulation amplitude = 10 Gpp.

### Theoretical analysis of the EPR spectra

The experimental EPR spectra were simulated using the EasySpin software. A S = 1/2 Spin Hamiltonian was used $\hat{H} = \beta \vec{B} \cdot g \cdot \vec{S}$ where β is the Bohr magneton, $\vec{B}$ is the applied magnetic field, g is the spectroscopic g-tensor and $\vec{S}$ the spin angular momentum. The X-band electron paramagnetic resonance (EPR) spectra were recorded with a Bruker ER200D spectrometer at 77 K, equipped with an Agilent 5310A frequency counter. The spectrometer was running under a home-made software based on LabView. Adequate signal-to-noise ratio was obtained after 30–50 scans. The EPR instrumental conditions were as follows: microwave frequency = 9.55 GHz, modulation frequency = 50.00 kHz, and modulation amplitude = 10 Gauss peak-to-peak.

### Photocatalytic H_2_ evolution procedure

The photocatalytic hydrogen reactions were realized into a double wall Pyrex reactor, cooled with tap circulation (T = 25 °C). Light source was a Solar Simulator, (Sciencetech, Class AAA, model SciSun-150) with average irradiation intensity of 180 W m^−2^ equipped with a xenon lamp of 150 W and Air Mass filter (1 sun, AM1.5G). As Visible light source was used a Led lamp FireEdge™ FE410 (λ = 405 nm) supplied by Phoseon company, which power intensity was set to be 180 W m^−2^, using a power meter (Thorlabs Inc., USA). In each experiment, 50 mg of the catalyst was suspended into 150 ml water/methanol mixture 20% v/v (final concentration of the catalyst 330 mg L^−1^). Atmospheric O_2_ from the suspension was removed, fulfilling the content of reactor with Ar gas (99.9997%) at least 1 h. As Pt source were used the dihydrogen hexachloroplatinate (IV) hydrate complex (H_2_Pt_4_Cl_6_. 6H_2_O, 99.99%, Αlfa Αesar) which was photodeposited in situ, at the reaction mixture. Qualitative and quantitative monitoring of produced H_2_ and CO_2_ gases was done via a continuous online GasChromatography System combined with a Thermo-conductive Detector (GC-TCD- Shimadzu GC-2014, carboxen 1000 column, Ar carrier gas^49^.

### Post-FSP oxidation

A ThermaWatt furnace was used, equipped with a tubular Quartz compartment^50^. Oxidations were performed under atmospheric O_2_ at temperature 400 °C and calcination time was varied from 30 to 120 min through intervals of 30 min.

## Supplementary Information

Supplementary Information.

## Data Availability

All data generated or analysed during this study are included in this published article (and its supplementary information files).
