# Supplementary material for: Control of monomeric Vo’s versus Vo clusters in ZrO2−x for solar-light H2 production from H2O at high-yield (millimoles gr−1 h−1)
Source: Sci Rep. 2022 Sep 7;12:15132. doi: 10.1038/s41598-022-19382-3 (PMC9452565; doi:10.1038/s41598-022-19382-3)
Supplement: Supplementary file 1 — Supplementary Information. [file 41598_2022_19382_MOESM1_ESM.docx]

*Supplementary Information for manuscript*

**Control of Monomeric Vo’s *vs.* Vo clusters in ZrO_2-x_ for Solar-Light H_2_ Production from H_2_O at High-Yield [millimoles gr^-1^ h^-1^]**

Yiannis Deligiannakis^1,4^*, Asterios Mantzanis^1^, Areti Zindrou^1^, Szymon Smykala^3^ and Maria Solakidou^1,2^

^1^ Laboratory of Physical Chemistry of Materials & Environment, Department of Physics, University of Ioannina, Ioannina, Greece

^2^ Laboratory of Biomimetic Catalysis and Hybrid Materials, Department of Chemistry, University of Ioannina, GR45110 Ioannina, Greece

^3^Institute of Engineering Materials and Biomaterials, Silesian University of Technology, 18a Konarskiego St, 44-100 Gliwice, Poland

^4^ Institute of Environment & Sustainable Development, University Research Center of Ioannina, GR45110 Ioannina, Greece

* Corresponding author: Yiannis Deligiannakis ([*ideligia@uoi.gr*](mailto:ideligia@uoi.gr)*)*

**Table S1.** FSP-process conditions for the synthesis ZrO_2_/ZrO_2-x_ materials.

| Materials | Dispersion O_2_ | Dispersion CH_4_ | P/D | Sheath O_2_(L/min) | Sheath N_2_(L/min) |
| --- | --- | --- | --- | --- | --- |
| [F.O.] | 3 | 0 | 3.0/3.0 | 10 | 0 |
| [3/0.1] | 3 | 0.1 | 3.0/3.1 | - | 10 |
| [3/0.2] | 3 | 0.2 | 3.0/3.2 | - | 10 |
| [2.3/0.7] | 2.3 | 0.7 | 3.0/3.0 | - | 10 |
| [1.3/1.7] | 1.3 | 1.7 | 3.0/3.0 | - | 10 |


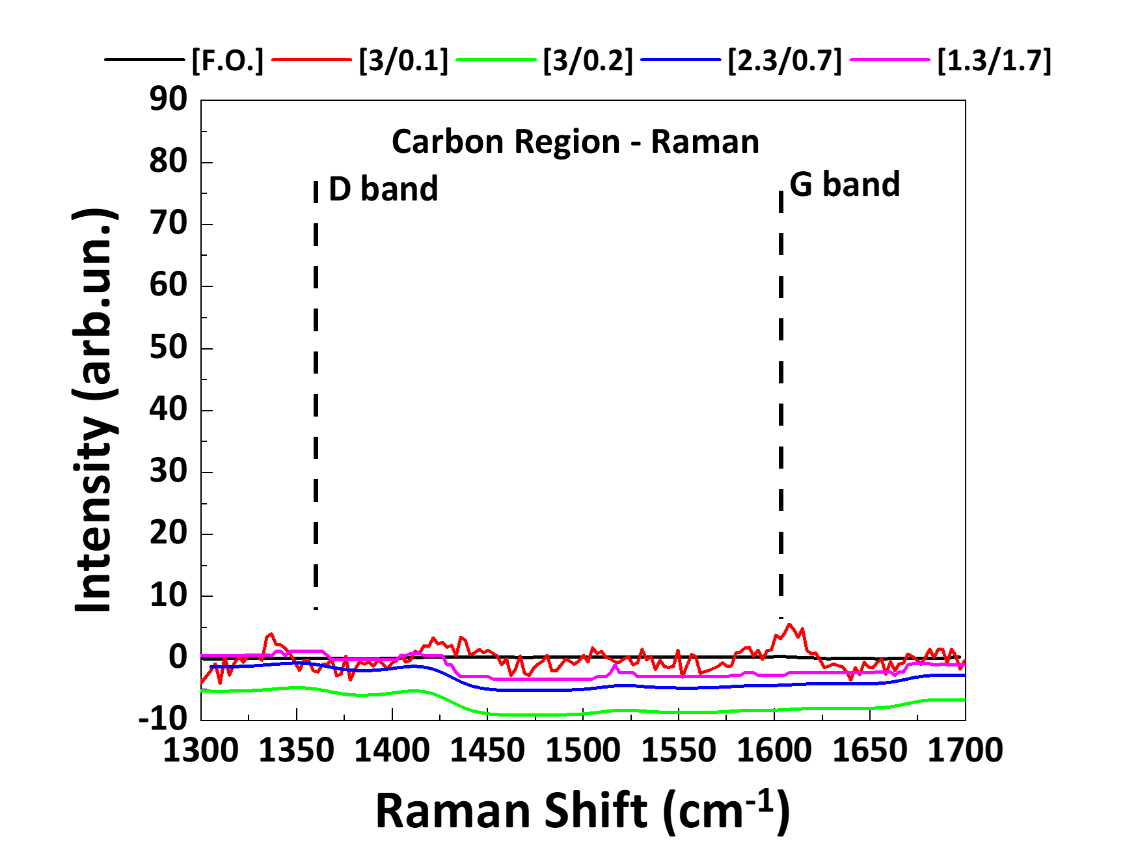


**Figure S1.** Raman spectra in the Carbon region, indicating the absence carbon-formation in the ΖrO_2-x_ materials made by FSP.


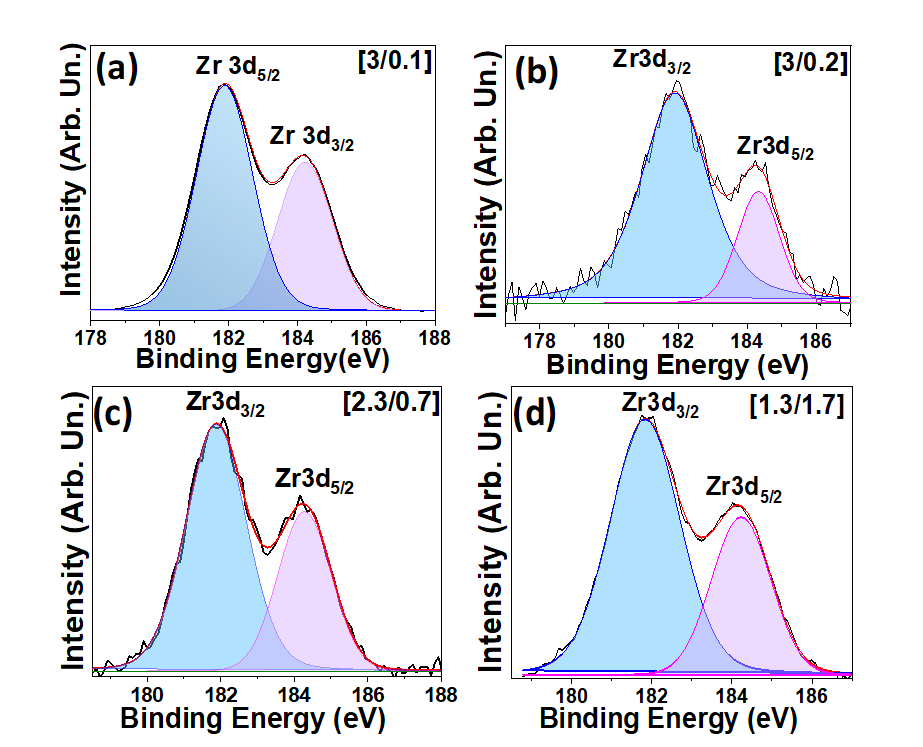
**Figure S2.** XPS spectra of the Zr doublet of (**a)** [3/0.1], **(b) [**3.0/0.2], **(c)** [2.3/0.7] and **(d)** [1.3/1.7].

**Table S2.** Structural characteristics of the present FSP-made ZrO_2-X_ materials.

| Materials | Calcination T/time  (℃)/(min) | Phase Comp.(%) | | Crystallite size (nm) | | | SSA (m^2^g^-1^) |
| --- | --- | --- | --- | --- | --- | --- | --- |
|  |  | m-ZrO_2_ | t-ZrO_2_ | d_XRD_ | | d_BET_ |  |
| **[F.O]** | - | 15 | 85 | 20 | 29 | 14 | 72 |
| **[3/0.1]** | - | 15 | 85 | 19 | 28 | 16 | 64 |
| [3/0.1]-60 | 400/60 | 30 | 70 | 18 | 26 | 17 | 58 |
| [3/0.1]-120 | 400/120 | 35 | 65 | 16 | 24 | 18 | 55 |
| **[3/0.2]** | - | 15 | 85 | 19 | 28 | 19 | 52 |
| [3/0.2]-30 | 400/30 | 25 | 75 | 19 | 27 | 20 | 50 |
| [3/0.2]-60 | 400/60 | 30 | 70 | 18 | 25 | 21 | 47 |
| [3/0.2]-90 | 400/90 | 30 | 70 | 17 | 24 | 22 | 45 |
| **[2.3/0.7]** | - | 15 | 85 | 18 | 24 | 23 | 44 |
| [2.3/0.7]-30 | 400/30 | 27 | 73 | 18 | 24 | 24 | 42 |
| [2.3/0.7]-60 | 400/60 | 30 | 70 | 17 | 23 | 25 | 40 |
| [2.3/0.7]-90 | 400/90 | 30 | 70 | 17 | 22 | 27 | 38 |
| [2.3/0.7]-120 | 400/120 | 33 | 67 | 16 | 22 | 27 | 37 |
| **[1.3/1.7]** | - | 15 | 85 | 17 | 25 | 29 | 34 |
| [1.3/1.7]-60 | 400/60 | 30 | 70 | 16 | 23 | 32 | 31 |

**Figure S3.** XRD patterns of **(a)** [3/0.1], **(b) [**3/0.2], **(c)** [2.3/0.7] and **(d)** [1.3/1.7] materials oxidized under ambient air at 400^0^C for the indicated time-periods (30,60,90,120 minutes).

**Figure S4.** Raman spectra of [3/0.1], [3/0.2], [2.3/0.7] and [1.3/1.7].

**Table S3.** Raman bond vibrations for t-ZrO_2_ and m-ZrO_2_.

| Tetragonal Zirconia Modes | | Monoclinic Zirconia Modes | | |
| --- | --- | --- | --- | --- |
| Assignment | R.Shift (cm^-1^) | Assignment | R.Shift (cm^-1^) | Bond Vibration^*^ |
| E_g_ | 145 | A_g_ | 100 | O-O |
| A_1g_ | 264 | A_g_ | 176 | Zr-Zr |
| B_1g_ | 313 | B_g_ | 176 | Zr-Zr |
| E_g_ | 460 | A_g_ | 186 | Zr-Zr |
| E_g_ | 643 | B_g_ | 220 | Zr-O |
| - | - | A_g_ | 270 | Zr-Zr |
| - | - | A_g_ | 305 | Zr-O |
| - | - | B_g_ | 330 | Zr-O |
| - | - | A_g_ | 345 | O-O |
| - | - | B_g_ | 381 | O-O |
| - | - | A_g_ | 472 | O-O |
| - | - | B_g_ | 502 | O-O |
| - | - | B_g_ | 536 | O-O |
| - | - | A_g_ | 554 | O-O |
| - | - | B_g_ | 616 | O-O |
| - | - | A_g_ | 633 | O-O |

**Table S4.** Raman shifts of ZrO_2-x_ materials.

| Materials | Mode (cm^-1^) | Shift (cm^-1^) | Assignment |
| --- | --- | --- | --- |
| [3.0/0.1] | 313 | +4 | Zr-Zr |
| [2.3/0.7] | 313 | -5 | - |
|  | 472 | -2 | - |
|  | 643 | -4 | - |
| [1.3/1.7] | 100 | -3 | O-O |
|  | 176 | -4 | Zr-Zr |
|  | 188 | -7 | - |
|  | 264 | -9 | Zr-Zr |
|  | 381 | -8 | O-O |
|  | 472 | -2 | O-O |
|  | 554 | -11 | O-O |
|  | 643 | -22 | - |


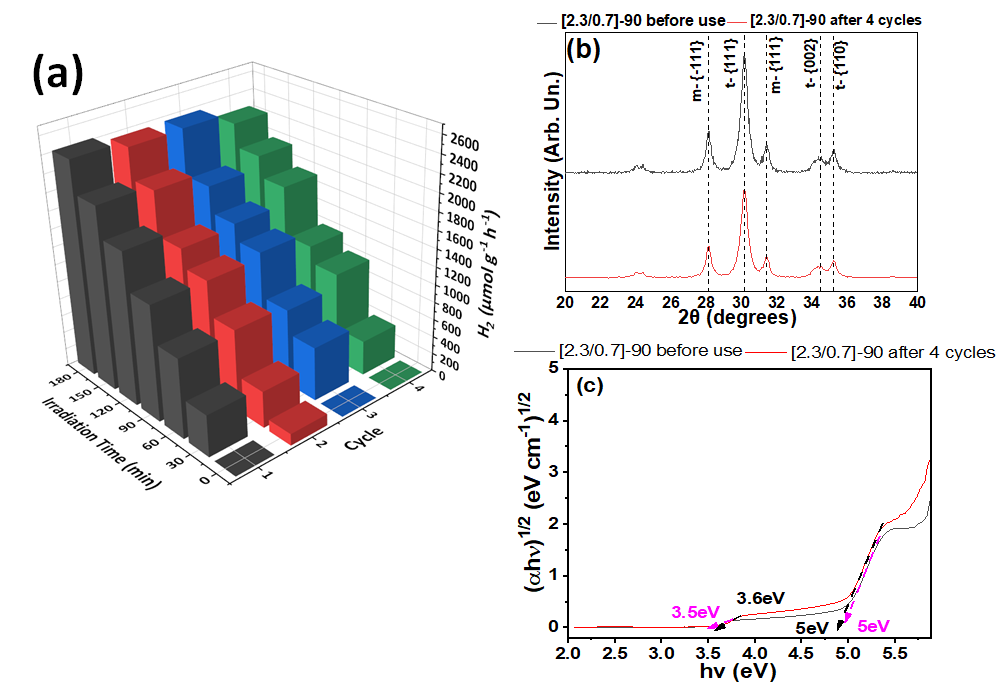
**Figure S5**. (**a**) Catalytic Reuse experiments of material [2.3/0.7]-90, (**b**) X-Ray Diffraction patterns and (**c**) DRS-UV/Vis spectra after 4 catalytic cycles.


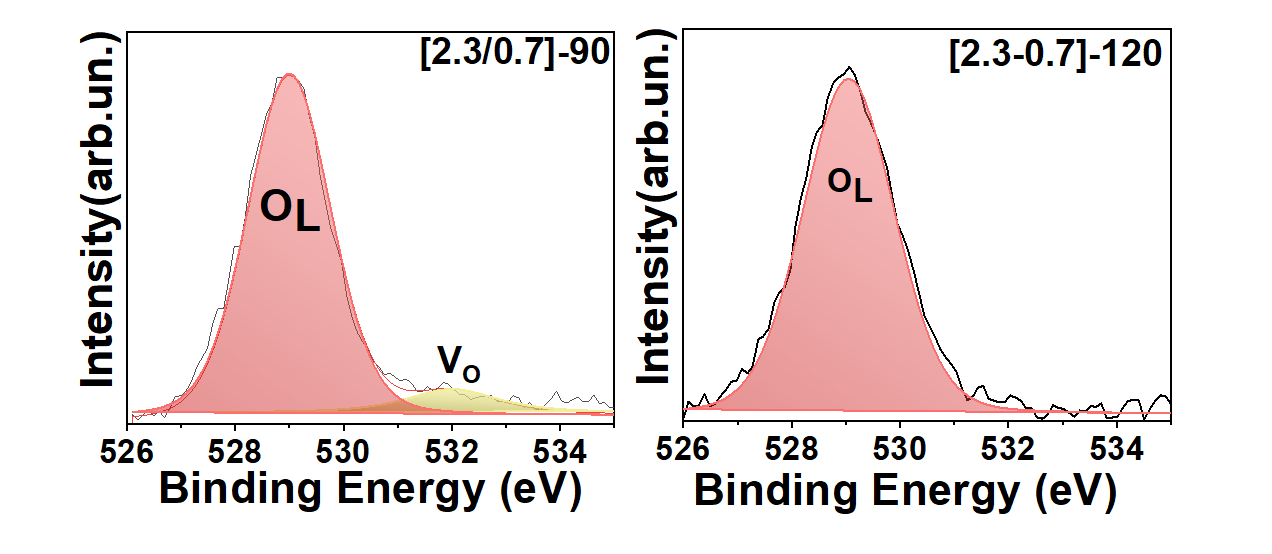


**(a)bn**

**(b)bn**

**Figure S6.** O1s XPS spectra of material [2.3/0.7] calcined for (**a**) 90 and (**b**) 120 min.

**Table S5**. Comparison of benchmark ZrO_2-x_ based photocatalysts in literature for H_2_ production

| **Materials** | **Source of Irradiation** | **Sacrificial Agent** | **Catalytic Yield** | **Ref.in SI** |
| --- | --- | --- | --- | --- |
| ZrO_2-x_ | | | | |
| ZrO_2-x_ [2.3/0.7]-90 | Solar Light 150W Xenon Lamp and AM 1.5G filter | 20 vol% MeOH – 1 wt% H_2_Pt_4_Cl_6_ 6H_2_O in-situ | 2428μmol g^-1^ h^-1^ | This work |
| Black ZrO_2-x_ (BZ) | Solar Light 100W Xenon Lamp and AM 1.5G filter | 20 vol% MeOH – 1 wt% H_2_Pt_4_Cl_6_ 6H_2_O in-situ | 505μmol g^-1^ h^-1^ | ^2^ |
| ZrO_2_-Black | Solar Light (100mWcm^-1^) with AM 1.5G filter | 20 vol% MeOH – 1 wt% H_2_Pt_4_Cl_6_ 6H_2_O in-situ | 506μmol g^-1^ h^-1^ | ^3^ |
| ZrO_2_ | High Pressure Hg Lamp (400W) | none | 72μmol g^-1^ h^-1^ | ^5^ |
| Heteroatom-ZrO_2_ | | | | |
| N-doped ZrO_2_ | 300W Xenon Lamp | 20 vol% MeOH – Chloroplatinic acid (1% Pt) | 2120μmol g^-1^ h^-1^ | ^1^ |
| ZrO_2_ – 3% Y_2_O_3_ | 300W Xenon Lamp with 400nm long-pass filter | 3 cm^3^ MeOH – 0.0513cm^3^ Pt (NH_3_)_4_(NO­_3_)_2_ | 160 μmol g^-1^ h^-1^ | ^4^ |


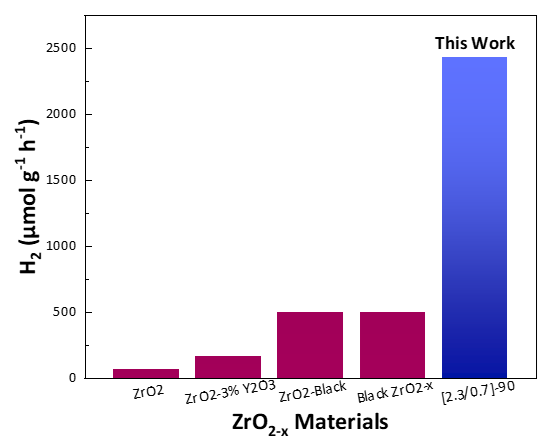
**Figure S7.** Comparison of photocatalytic H_2_ production by various ZrO_2-x_ & cases of Heteroatom-ZrO_2_ materials


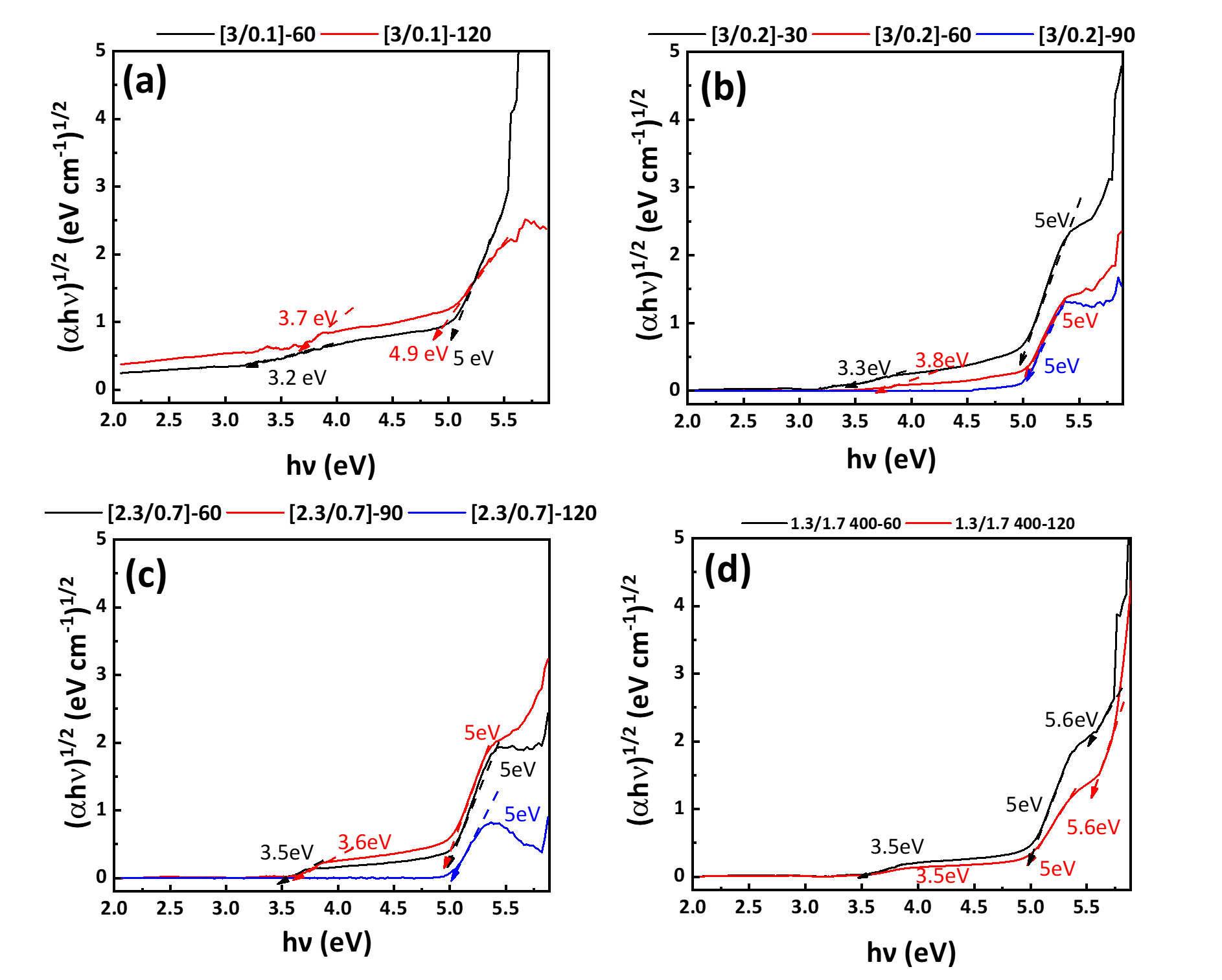
**Figure S8.** UV-Vis/DRS spectra of calcined **(a)** [3/0.1], **(b) [**3.0/0.2], **(c)** [2.3/0.7] and **(d)** [1.3/1.7] materials.

**Table S6**. EPR parameters for Vo signals in, ([3/0.1], [3/0.2], [2.3/0.7] and [1.3/1.7]).

| Material | Monomer | | | ΔH(±0.1G) | Cluster | ΔH(±0.1G) |
| --- | --- | --- | --- | --- | --- | --- |
|  | g_zz_ | g_yy_ | g_xx_ |  | g_iso_ |  |
| 3/0.1 | 2.0072 | 2.0037 | 2.0011 | 5 | - | - |
| 400-60 | 2.0059 | 2.0042 | 2.0020 | 6 | - | - |
| 400-120 | 2.0060 | 2.0046 | 2.0034 | 5.2 | - | - |
| 3/0.2 | 2.0072 | 2.0037 | 2.0011 | 4.8 | 2.0028 | 6.5 |
| 400-60 | 2.0078 | 2.0041 | 2.0008 | 6.8 | - | - |
| 400-90 | 2.0076 | 2.0040 | 2.0007 | 8.6 | - | - |
| 400-120 | 2.0060 | 2.0043 | 2.0038 | 5.1 | - | - |
| 2.3/0.7 | 2.0072 | 2.0037 | 2.0011 | 4.8 | 2.0028 | 6.5 |
| 400-60 | 2.0072 | 2.0037 | 2.0011 | 4.8 | 2.0028 | 6.5 |
| 400-90 | 2.0078 | 2.0045 | 2.0020 | 5.8 | - |  |
| 400-120 | 2.0072 | 2.0041 | 1.9993 | 5.3 | - |  |
| 1.3/1.7 | 2.0072 | 2.0037 | 2.0011 | - | 2.0028 | 6.5 |
| 400-60 | 2.0072 | 2.0037 | 2.0011 | 4.8 | 2.0028 | 6.5 |
| 400-90 | 2.0072 | 2.0037 | 2.0011 | 4.8 | 2.0028 | 6.5 |
| 400-120 | 2.0072 | 2.0037 | 2.0011 | 4.8 | 2.0028 | 6.5 |


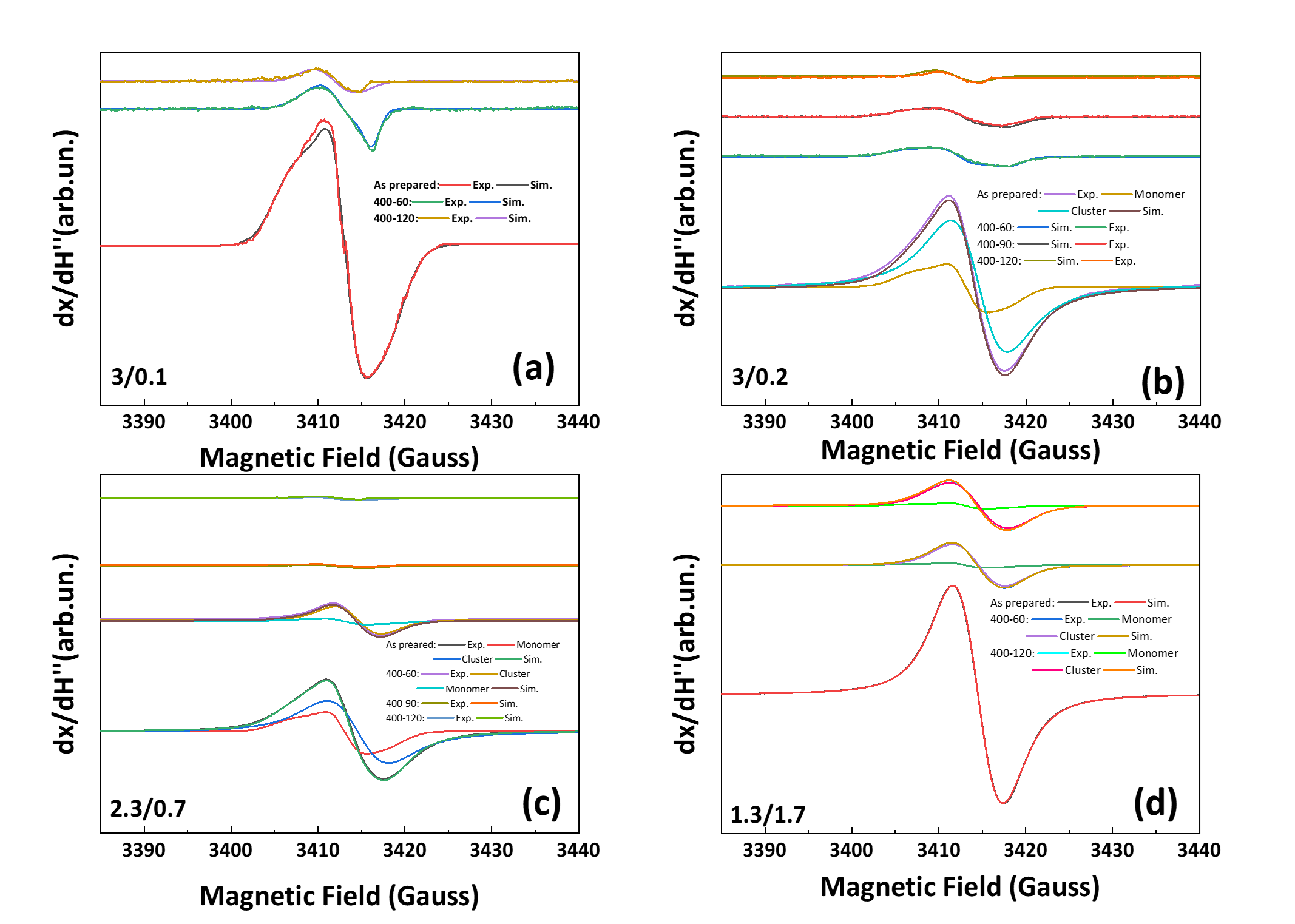
**Figure S9**. Experimental and fitted EPR spectra of (**a**) [3/0.1] calcined at 400 $℃$ for 60min and 120min, (**b**) [3/0.2] calcined at 400 $℃$ for 60min, 90min and 120min, (**c**) [2.3/0.7] calcined at 400 $℃$ for 60min, 90min and 120min and (**d**) [1.3/1.7] calcined at 400 $℃$ for 60 min and 120 min.

**Table S7**. EPR Quantitation of V_O_ centers in μmolg^-1^ of [3/0.1], [3/0.2], [2.3/0.7], [1.3/1.7] and calculation of V_O_ / Zr atom ratio.

| Materials | μmolg^-1^ x10^3^ (V_O_) | V_O_/Ζr-atom (x10^4^) |
| --- | --- | --- |
| [3/0.1] | 21 | 22 |
| [3/0.2] | 24 | 25 |
| [2.3/0.7] | 32 | 35 |
| [1.3/1.7] | 148 | 159 |

**Theoretical DFT Calculations:** Theoretical calculations were conducted for the t-ZrO_2_ phase, using DFT as implemented in the Quantum Espresso software^6^. The projector augmented-wave (PAW) method was used to describe the core pseudopotential and the generalized gradient approximation (GGA) as simplified by Perdew, Burke, and Ernzerhof (PBE) was used for exchange-correlation effects. Ultrasoft pseudopotentials containing 12 valence electrons were used for Zr ([Kr] 5s2 5p0 4d2) and 6 valence electrons for O ([He] 2s2 2p4). A plane-wave energy cutoff of 45.551 Ry was used. Using the B.F.G.S routine, the structure was relaxed until the forces acting on each atom were <10^-3^ meV/Å. For geometry optimization and electronic structure calculations, (1 × 1 × 2) and (2 × 2 × 5) Monkhorst-Pack k-point grids were used respectively. Considering the strong correlations in transition metal oxides, we used the DFT+U approximation in the approach of Cococcioni and de Gironcoli^7^ with a U-value of 4 eV applied on all structures.


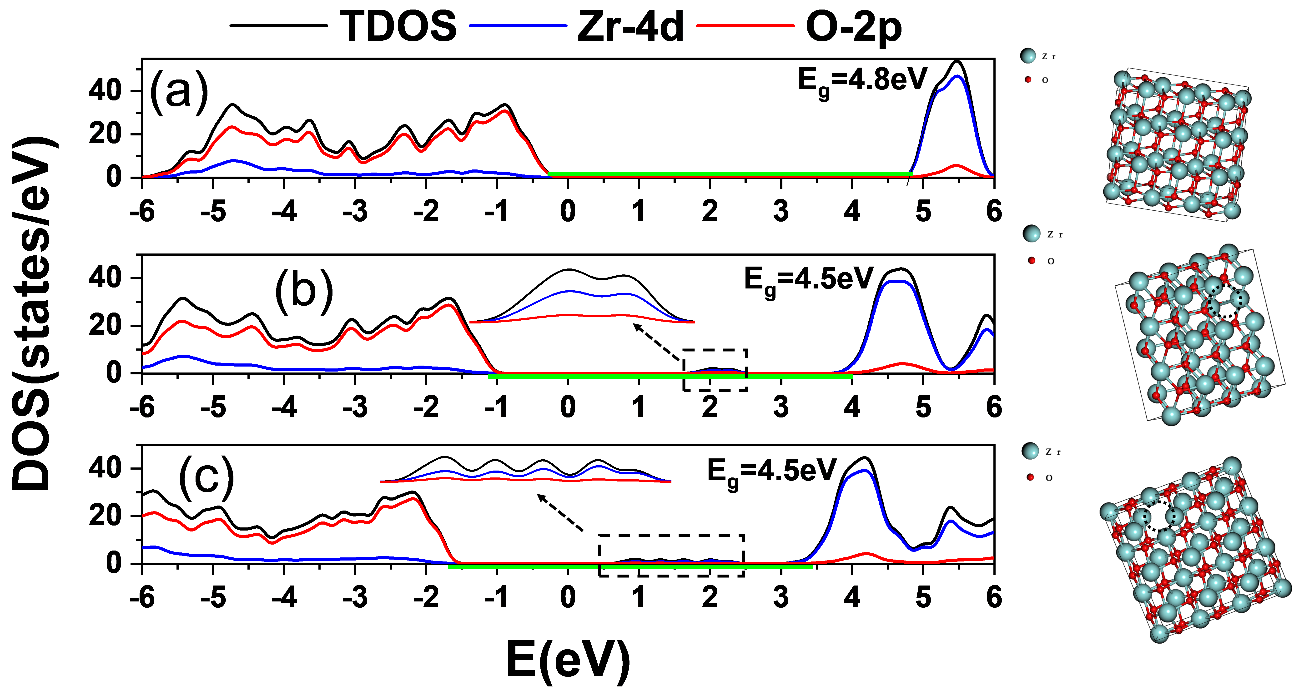


**Figure S10**. Theoretical DFT calculated electron Density of States for (**a**) ZrO_2_, (b) ZrO_2_-(with one Vo per 9-unit cells) and (**c**) ZrO_2_-(with two Vo per 9-unit cells). The green-bar in (**a**) marks the Eg-value for pristine ZrO_2_. In (**b**), (**c**) is re-marked to facilitate the visualization of the band-gap narrowing and the creation of DOS tail in the CB-bottom. (Right panels) the 9-unit cell structures used to calculate the DOS by DFT.

**References:**

1. Wang, Y. *et al.* Novel N-doped ZrO 2 with enhanced visible-light photocatalytic activity for hydrogen production and degradation of organic dyes. (2018) doi:10.1039/c7ra12938f.

2. Sinhamahapatra, A., Jeon, J. P., Kang, J., Han, B. & Yu, J. S. Oxygen-Deficient Zirconia (ZrO2−x): A New Material for Solar Light Absorption. *Sci. Reports 2016 61* **6**, 1–8 (2016).

3. Zu, D. *et al.* Black ZrO2 synthesized by molten lithium reduction strategy for photocatalytic hydrogen generation. *J. Am. Ceram. Soc.* **103**, 4035–4042 (2020).

4. Wang, Q. *et al.* Photocatalytic hydrogen generation on low-bandgap black zirconia (ZrO2 ) produced by high-pressure torsion. *J. Mater. Chem. A*, 2020,8, 3643-3650 (doi:10.1039/c9ta11839j).

5. Sayama, K. & Arakawa, H. Physical Chemistry &copy. **97**, (1993).

6. Browning, R. *et al.* QUANTUM ESPRESSO: a modular and open-source software project for quantum simulations of materials You may also like Carrier conductance in 2D WSe 2 films The status of varying constants: a review of the physics, searches and implications C J A P Martins-Revisiting WASP-47 with ESPRESSO and TESS QUANTUM ESPRESSO: a modular and open-source software project for quantum simulations of materials. *J. Phys. Condens. Matter* **21**, 395502 (2009).

7. Cococcioni, M. & De Gironcoli, S. Linear response approach to the calculation of the effective interaction parameters in the LDA+ U method. doi:10.1103/PhysRevB.71.035105.
